# Expanded Screening of One Million Swedish Babies with R4S and CLIR for Post-Analytical Evaluation of Data

**DOI:** 10.3390/ijns6020042

**Published:** 2020-05-27

**Authors:** Lene Sörensen, Ulrika von Döbeln, Henrik Åhlman, Annika Ohlsson, Martin Engvall, Karin Naess, Carolina Backman-Johansson, Yvonne Nordqvist, Anna Wedell, Rolf H. Zetterström

**Affiliations:** 1 Centre for Inherited Metabolic Diseases, Karolinska University Hospital Solna, SE-171 76 Stockholm, Sweden; ulrika.vondobeln@sll.se (U.v.D.); henrik.ahlman@sll.se (H.Å.); annika.ohlsson@eskilstuna.se (A.O.); martin.engvall@sll.se (M.E.); karin.naess@ki.se (K.N.); carolina.backman-johansson@sll.se (C.B.-J.); yvonne.m.nordqvist@sll.se (Y.N.); anna.wedell@ki.se (A.W.); rolf.zetterstrom@sll.se (R.H.Z.); 2 Department of Molecular Medicine and Surgery, Karolinska Institutet, SE-171 76 Stockholm, Sweden; 3 Department of Medical Biochemistry and Biophysics, Division of Molecular Metabolism, Karolinska Institutet, SE-171 77 Stockholm, Sweden

**Keywords:** newborn screening, post-analytical evaluation, Collaborative Laboratory Integrated Reports (CLIR), tandem mass spectrometry, expanded screening, dried blood spots (DBS), positive predictive value (PPV)

## Abstract

Sweden has one neonatal screening laboratory, receiving 115 to 120 thousand samples per year. Among the one million babies screened by tandem mass spectrometry from November 2010 until July 2019, a total of 665 babies were recalled and 311 verified as having one of the diseases screened for with this methodology, giving a positive predictive value (PPV) of 47% and an incidence of 1:3200. The PPV was high (41%) already in the first year after start of screening, thanks to the availability of the collaborative project Region 4 Stork database. The PPV is presently 58%. This improvement was achieved by the implementation of second-tier analyses in the screening for methylmalonic aciduria, propionic aciduria, isovaleric aciduria, and homocystinuria, and the employment of various post analytical tools of the Region 4 Stork, and its successor the collaborative laboratory integrated reports.

## 1. Introduction

With the emergence of tandem mass spectrometry (MS/MS) technology during the 1990s [1,2], the number of inborn errors of metabolism (IEMs) that could be included in dried blood spots (DBS) screening programs increased several-fold [3,4,5,6,7,8,9].

Sweden introduced newborn screening for phenylketonuria (PKU) using DBS in 1965 [10,11]. Between 1967 and 2002, four additional diseases were included in the program: galactosemia (1967), congenital hypothyroidism (1980), congenital adrenal hyperplasia (1986), and biotinidase deficiency (2002). In 2005, the Swedish newborn screening laboratory introduced MS/MS technology for PKU screening [12] and 19 additional inborn errors of metabolism (IEM) were included in the program on November 15, 2010 [11]. After the addition of severe combined immunodeficiency in 2019, 25 diseases are presently included in the national screening program. Compliance with the program is very high, with a participation of more than 99.5% of newborn babies nation-wide. Presently between 115 and 120 thousand babies are born in Sweden per year [13]. 

In 2004, seven U.S. states (Illinois, Indiana, Kentucky, Michigan, Minnesota, Ohio, and Wisconsin) initiated a laboratory quality improvement project called Region 4 Stork (R4S). The goal was to improve the performance of expanded newborn screening by MS/MS. This was accomplished by facilitating laboratory data sharing between different screening laboratories and providing tools for the interpretation of local screening data [14,15,16]. The Swedish screening laboratory joined the R4S initiative before the introduction of expanded screening with MS/MS. Between 2010 and 2012, six members of the Swedish team attended a one week “training program for newborn screening by MS/MS” at the Mayo Clinic in Rochester, Minnesota. 

In September 2018, the R4S website was replaced by Collaborative Laboratory Integrated Reports (CLIR) [17]. In addition to laboratory data, CLIR also takes covariate data, such as age at sampling and birth weight, into account when calculating screening result scores [18]. The Swedish laboratory started to use CLIR in October 2018. Since 2017, members from our laboratory have attended several CLIR collaborator workshops as well as a bi-weekly teleconference “CLIR collaborator call”. 

The scope of this article is to report the outcome of expanded screening by MS/MS technology of one million Swedish newborn babies and the improved results obtained using R4S/CLIR and second-tier testing.

## 2. Materials and Methods

### 2.1. Study Subjects

One million Swedish babies were screened from 15 November 2010 through 1 July 2019, with MS/MS technology. Blood was collected on Perkin Elmer 226 Ahlstrom filter paper, usually from a vein on the back of the hand. The sample was collected as soon as possible after 48 h from birth. Median age at sampling was 57 h and the 90th percentile was at 92 h. Families received written information in the third trimester, oral information at the time of sampling, and a second written document was given to the parents when the sample was taken. Only oral informed consent was requested.

### 2.2. Screening Laboratory Work-Flow

Sweden has one centralized laboratory for the DBS newborn screening program, located at the Centre for Inherited Metabolic Diseases at Karolinska University Hospital in Stockholm. Samples arrive to the laboratory by regular mail or in some cases via a hospital-run transport network. 

**Figure 1:**
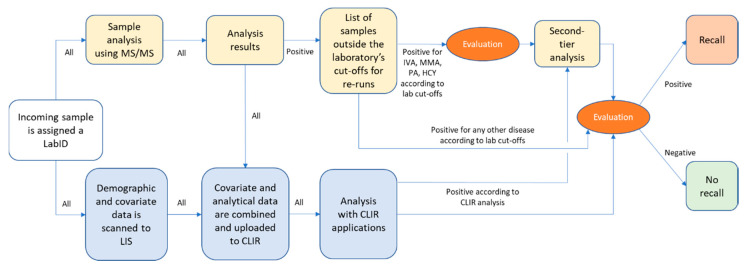
Flow-chart of the present work-flow for MS/MS screening in the Swedish newborn screening laboratory. A list of samples outside laboratory cut-offs and CLIR analysis is combined and evaluated for recalls. In some cases (IVA, MMA, PA, HCY) second-tier analysis is performed before the recall decision is made.

The filter paper with the blood sample and the connected referral notes are assigned identical laboratory identification numbers before being separated into two parts; one sample part and one referral part containing all demographic and covariate data. While the sample is analyzed with MS/MS, the demographic and covariate data are scanned into the laboratory information system (LIS; Figure 1). 

All samples, in which the initial analysis result is outside the laboratory cut-off, are re-run in duplicate before final evaluation. Since mail is delivered only Monday to Friday, the screening laboratory is routinely not in operation during weekends or public holidays. All samples arriving on Fridays are therefore analyzed and re-run on the same day. To avoid a delay of time-sensitive recalls over the weekend, methylmalonic aciduria (MMA) and propionic aciduria (PA) recalls on Fridays may be made before second-tier analysis is completed. 

The laboratory defines a “recall” as any contact made with the family, prompted by a suspicion that the child might have one of the screened diseases. Repeat samples were requested when the screening result was abnormal, pointing to ongoing total parenteral nutrition (TPN) treatment, or when the initial sample was of bad quality. These repeat samples are not included in the recall numbers. Among the one million babies there were 1178 repeat samples due to TPN treatment and 2547 due to bad quality of the initial sample.

### 2.3. MS/MS Screening Method

The MS/MS screening uses the NeoBase™ screening kit from Perkin Elmer (Waltham, MA, USA). Between 2010 and 2015 two Quattro Micro™ and one Quattro Premier™ from Waters (Milford, MA, USA) were used. In 2015 and 2016 these were exchanged with three Xevo^®^ TQDs. Specimen Gate^®^ MSMS Datasuite from Perkin Elmer, were employed for collection of data. The result of the first analysis is available on the same day and re-runs of elevated or failed analyses are available either the same or the following day. 

After analysis, results and covariate data were combined and temporarily uploaded to CLIR for evaluation [17]. Before October 2018, results, without covariate data, were uploaded to R4S for evaluation.

Second-tier testing was performed on either a Xevo^®^ TQ-S, Xevo^®^ TQ-MS, or a Xevo^®^ TQ-XS from Waters. A protocol for measurement of methionine, homocysteine, methylmalonate, and methylcitrate has been published previously [19]. We used a modified version of this protocol. It takes two days before the result is ready. A method for the separation of C5 isomers (pivaloylcarnitine, isovalerylcarnitine and 2-methylbutyrylcarnitine) was developed in our laboratory. It employs a simple separation step, enabling a very quick (approximately one hour) qualitative analysis. A similar method has been published later by others [20].

### 2.4. Evaluation of Screening Data

When preparing the implementation of MS/MS DBS screening in 2010, we established cut-off values for each disease with the help of R4S data [14]. These values were used for re-runs in duplicate, followed by evaluation, except for PKU where we already had established cut-offs [12]. Over the years we have increased the use of R4S/CLIR tools for recall decisions (Figure 1). 

For isovaleric aciduria (IVA), MMA, PA, and homocystinuria (HCY) second-tier tests are performed, in addition to the first analysis, before a decision to recall is made (Figure 1) [19,20,21]. 

The decision to recall is made after a joint evaluation of the laboratory cut-off levels and R4S/CLIR results (Figure 1). Recall is made by phone to a pediatrician at one of four metabolic centers, followed by a written report.

### 2.5. Confirmation of True or False Cases and Feedback on Missed Cases

Diagnostic samples and a second DBS for confirmation are taken, along with a clinical evaluation of the infant at one of the metabolic treatment centers or at the home hospital, depending on the situation. The purpose of the second DBS is to compare analyte levels at recall with the levels at screening with identical analytical methods, and to give the screening laboratory a verification that the child has been recalled. 

Biochemical and genetic confirmatory analyses are performed at one of two specialized metabolic laboratories in Sweden; Centre for Inherited Metabolic Diseases at Karolinska University Hospital, of which the screening laboratory is a section; and The Department of Clinical Chemistry at Sahlgrenska University Hospital in Gothenburg. The results are communicated back to the screening laboratory. The Swedish IEM community has meetings twice a year, where screening results and newly diagnosed patients are discussed.

### 2.6. Data Collection and Analysis

The screening laboratory has a comprehensive database containing all samples, recalls, and information about true and false positive cases. For the present report, we made a query for all recalls including sample arrival date, reason for recall, if it was true or false, and comments (e.g., known maternal medication). We also made a second query with only the numbers of incoming samples during specific time periods e.g., before or after implementation of second-tier analysis. No personal information, apart from diagnosis and the date of arrival at the laboratory of the first screening sample from each baby, was included.

### 2.7. Compliance with Ethical Standards

This study was approved by the Swedish Ethical Review Authority, approval number 2019-05816, approval date 18 December 2019.

## 3. Results

Among the first million babies screened with MS/MS technology after the extension from five to 24 disorders, 311 children were found and later diagnosed as true positive cases. We included cases of vitamin B12 deficiency among the true MMA/PA positive cases (Table 1). With 665 recalls, this gives an overall positive predictive value (PPV) of 47%. During the first year the PPV was 41%, and during the last year it was 58%. The overall incidence of the included diseases during the reporting period was 1:3200. 

The PPV of the different diseases varies, from 100% for betaketothiolase (BKT) deficiency to 12% for carnitine uptake deficiency (CUD), and the incidences vary from 1:500,000 (arginase deficiency (ARG) and BKT) to 1:14,000 (PKU). The PKU group includes classical PKU, hyperphenylalaninemia (HPA), and cofactor deficiencies (Table 1). Medium-chain acyl-coenzyme A dehydrogenase (MCAD) deficiency is almost as common as PKU, with an incidence of 1:17,000. 

One of the most complex diseases to screen for is CUD. In the Swedish screening program, with the help of R4S/CLIR, 13 children with CUD were found among these first million babies screened. With 94 false positives during the same time period, the PPV was 12% (Table 1).

Sweden has only two specialized diagnostic laboratories for IEMs, which enabled us to sum up the number of clinically diagnosed cases during the 20 years preceding the start of expanded screening. The incidences for some of the diseases, among approximately 2.12 million babies born, were thus determined (Table 1).

The incidences were similar before and after the screening was introduced (Table 1) for the following diseases: HCY, maple syrup urine disease (MSUD), PKU, glutaric aciduria type 1 (GA1), long-chain 3-hydroxyacyl-coenzyme A dehydrogenase deficiency (LCHAD) including mitochondrial trifunctional protein deficiency, tyrosinemia type 1 (TYR), and ARG. One case each of ARG deficiency and citrullinemia type 1 (CIT) had been diagnosed during these 20 years. It must be pointed out that the frequencies of the rarest disorders are estimated from very low numbers (1–3 cases; Table 1). 

For other diseases there was a striking increase of incidence, e.g., MCAD and very long-chain acyl-coenzyme A dehydrogenase deficiency (VLCAD; Table 1). This can partly be caused by poorer diagnostic conditions during the period of 20 years prior to start of screening.

Only four missed cases are known, all before early 2016. The missed cases included one patient with argininosuccinic aciduria (ASA) from 2016, two patients with carnitine palmitoyltransferase 2 (CPT2) deficiency from 2011 and 2012, respectively, and one patient with PA from 2014 (Table 1). Of these, two would have been found with the CLIR post-analytical tools, whereas two, a mild myopathic CPT2 and the PA case, would not.

The sensitivity of the screening of the first one million babies in Sweden was 98.73% and the specificity was 99.96%. The false positive rate was 0.035% and the false negative rate 1.27%.

**Figure 2:**
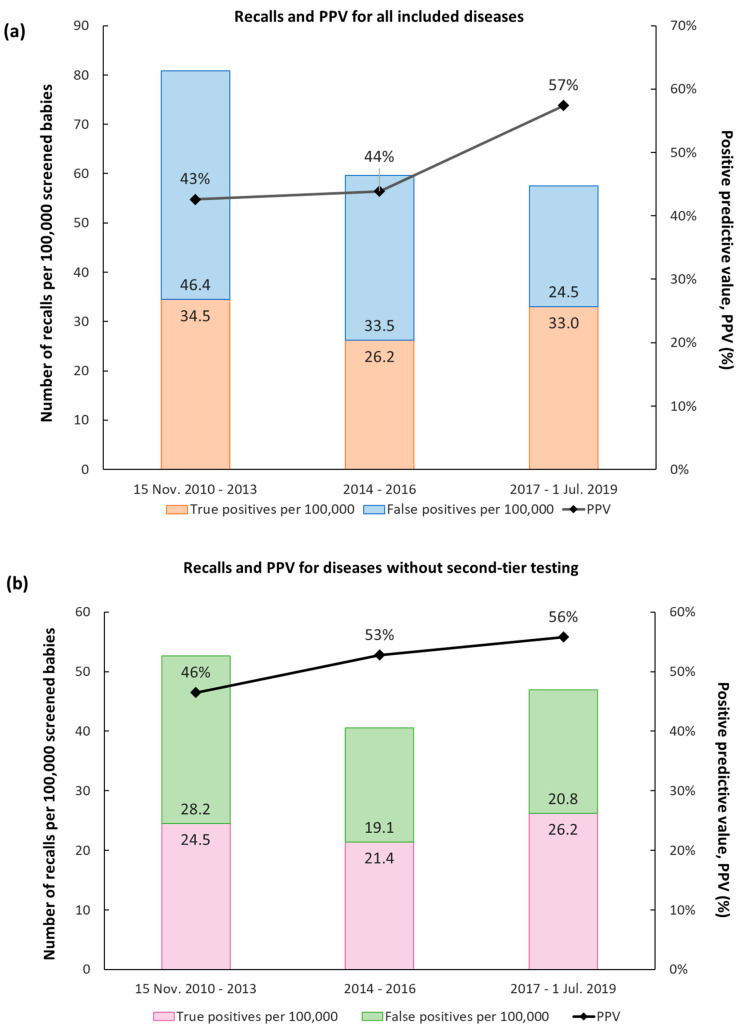
Recalls divided into three periods representing three different phases of the expanded screening program. Phase 1: 15 November 2010–2013; Introducing second-tier analysis for IVA; Using Singe Condition Tool in R4S; 351,211 babies screened. Phase 2: 2014–2016; Introducing Tool Runner and Dual Scatter Plot in R4S; 355,332 babies screened. Phase 3: 2017–1 July 2019; Introducing second-tier analyses for HCY, MMA, PA; Start using CLIR; 293,791 babies screened. During the whole period 1,000,334 newborns were thus screened, and the overall positive predictive value was 47%. (**a**) All recalls; (**b**) Recalls for all diseases except those where a second-tier analysis was implemented, i.e., all but MMA, PA, HCY, and IVA.

The Swedish MS/MS DBS screening program, from the start until the end of the reporting period, can roughly be divided into three phases. The first phase, 2010–2013, was a start-up phase, during which there were more false positive cases. The R4S Single Condition Tool was used from the very beginning. The second tier for IVA was developed very early on, less than a month from start. The overall PPV was 43% and the total recall rate was 80 per 100,000 newborns (Figure 2a). 

During the second phase, 2014–2016, we started to routinely employ more R4S post-analytical tools, such as Single Condition Tools and Dual Scatter Plot through the Tool Runner functionality. 

The PPV was similar at 44% but the recall rate dropped to just under 60 per 100,000 newborns, mainly due to a lower recall rate of babies with B12 deficiency (Figure 2a).

The third phase was from 2017 until the end of the reporting period, 1 July 2019. During this period, we employed second-tier analyses for HCY, MMA, PA, and the CLIR post-analytical tools, which improved the PPV to 57%. The overall recall rate remained roughly the same. Thus, the improved PPV observed is caused by a larger proportion of true positive cases (Figure 2a). 

A second-tier analysis for IVA was developed very early and was implemented after just over 7000 babies had been screened. Before that, there was a high number of false positives (and no true positive cases) for IVA due to maternal treatment with pivalinic acid containing antibiotics. After implementation of the second tier, there has only been one false positive recall, resulting in a PPV of 86% (Table 2). 

In January 2017, we implemented second-tier analyses for HCY and MMA/PA, respectively. There have not been any false positives for HCY since then, giving a PPV of 100%. At the same time, the PPV for MMA/PA increased almost two-fold from 34% to 62% (Table 2).

Leaving out the diseases for which we have a second-tier analysis, thus showing the effect of improved analytical performance and post-analytical evaluation, the increase in PPV from phase one to three was 1.22 times (from 46% to 56%; Figure 2b).

## 4. Discussion

We were relatively late to implement expanded screening with MS/MS in Sweden. Before expansion, candidate diseases were evaluated, taking the Wilson–Jungner criteria and published reports into account [3,4,5,6,7,8,22,23]. Which diseases to include was also discussed between experts in the field of mass screening and pediatricians, especially those at the four metabolic centers involved in the treatment of IEMs. We included 19 new disorders in the screening program (Table 1). Short-chain acyl-coenzyme A dehydrogenase deficiency and 3-methylcrotonyl-coenzyme A carboxylase deficiency were not included due to questionable indication for treatment. We joined the R4S collaborative project before the start of the expanded screening, giving us the opportunity to set our cut-off levels with the help of tools and reports available from the R4S website. With support from data supplied by R4S, our PPV values were very high (41%) already in the first year.

During the study period, there was a continuous improvement of the PPV, from 41% to 58% during the first and last year respectively. This represents a total improvement of around 40%. The development of a second-tier analysis for IVA, already during the first month, was instrumental for keeping the PPV of IVA and the total expanded screening at a high level (Table 2). With eleven false positive recalls after only 7300 screened, we would have had an untenable number of false positive recalls, an outcome we prevented by setting up the method rapidly (Table 2). The implementation of a second-tier analysis for MMA/PA, in January 2017, reduced the incidence of false positive recalls close to four times (Table 2). The high incidence of this disease group had great impact on the PPV for the whole expanded screening in the third period 2017–2019 (Figure 2a,b). The second-tier analysis for HCY, implemented in January 2017, resulted in a rise in PPV from 8% to 100%, so far, but the number of recalls has been low and has therefore not had much impact on the PPV for the screening as a whole (Table 2).

For the other diseases, the PPV has increased steadily as the R4S and CLIR tools were used more extensively (Figure 2b). When we started to evaluate the data from every single child in the R4S Tool Runner and other R4S tools, such as Dual Scatter Plot in January 2014, the rate of false positive as well as true positive recalls decreased while the PPV increased (Figure 2b). In the last phase, the PPV increased even further. During this period, we also transitioned from R4S to CLIR (Figure 2b). For this group of disorders, the number of true positive recalls is now 1.25 times that of false positive ones, which is very high. To our knowledge, there have not been any missed cases since 2017 until now (April 2020).

Homocystinuria is worth commenting on. The best result of treatment is seen in the pyridoxin responsive variants of the disease [24]. Several such cases are, however, missed by neonatal screening [25]. Early diagnosis and implementation of treatment through newborn screening has improved the prognosis in the severe variants of HCY [25,26]. In Sweden, the incidence seems to have been essentially equal before and during screening (Table 1). The first three patients identified by screening have not been responsive to treatment with pyridoxin. During recent years, we have diagnosed an increasing number of young adults with pyridoxine responsive HCY, probably due to better awareness of the disease. The future will tell how many infants with pyridoxine responsive HCY we have missed.

We do not have the exact numbers of cases, diagnosed clinically, with defects of the carnitine cycle before start of the screening, but they have been less than ten. The high incidence of CUD, along with a high incidence of false positive screening results, has been discussed by others [9,27,28]. In our cohort, six of the 94 false positive cases were verified by genetic analysis to be caused by CUD in the mothers, none of which had any symptoms (Table 1). In an interesting article, Dr Webster’s group presents important arguments for their decision to stop screening for CUD in New Zeeland [29]: low PPV levels, high rate of identified mothers without symptoms, dangers with unnecessary treatment with L-carnitine repressing the internal synthesis of carnitine, and long-term risks from the overproduction of the possibly carcinogenic metabolite trimethylamine-N-oxide from carnitine. The fact that one of the approximately three clinically diagnosed cases of CUD in Sweden died of liver failure and cardiac arrest and had fat deposition in heart and liver and the present quality of our CUD screening has however led to the decision to continue screening for CUD in Sweden.

Medication with pivalinic acid containing antibiotics, which reduces carnitine levels [30], is frequent in pregnant women in Sweden and was the cause of false positive CUD recalls in six babies. The carnitine lowering effect of maternal CUD and pivalinic acid may cause an increased risk of missing some infants with IEMs [31] but this has not been observed by us.

During the early nineties, there was an increasing awareness of MCAD deficiency as a potentially lethal disease. We investigated the frequency of the most common ACADM variant; 985A>G, at that time representing over 90% of the variants identified in clinically diagnosed patients with MCAD deficiency [32]. Eight heterozygotes were found by analysis of 1015 anonymous DBS samples, giving an estimated incidence of homozygosity for the variant of 1:65,000 newborn babies, to be compared with the incidence of 1:236,000 found clinically during the 20 years before screening (Table 1) [33]. Our incidence of 1:17,000 infants by screening is close to what has been found in other countries by screening [4,5,6,8,9,34]. It is obvious that we all find babies who will never develop symptoms, but we still do not know which ones.

In our screened cohort, VLCAD deficiency had an incidence of 1:43,000, as opposed to only one per million during the preceding 20 years (Table 1). Our current incidence is high, also in comparison with screening results from other countries [4,5,6,8,9,34]. The PPV is also high (Table 1), supported by the R4S/CLIR tools, but we know that attenuated forms of the disorder are frequent, resulting in a high risk of over-treatment. This dilemma can be solved, to some extent, with measurements of the enzymatic activity and determination of disease-causing genetic variants [35], but these results are not available before the initial treatment has started. 

The low positive predictive value of multiple acyl-coenzyme A dehydrogenase (MAD) deficiency is problematic. For verification of recalled cases, we employ whole genome sequencing focused on the genes involved in the generation of the cofactor system for the acyl-coenzyme A dehydrogenases [36,37]. Several Swedish patients, verified to have inherited defects causing MAD deficiency, and in need of treatment, have been missed before the introduction of newborn screening. Only a few cases (exact numbers not known) had been diagnosed clinically. 

Every false positive recall disturbs a family during a sensitive time and should be avoided when possible [38,39]. For extremely rare diseases, the number of false positives, even with a low PPV, will be small. Elevating the PPV for a more common disease thus prevents unnecessary distress in more families. With our present PPV, 58%, we have less than one false positive per true positive case, even though some (e.g., CUD) PPVs are low (Table 1).

## 5. Conclusions

Expanded screening of one million Swedish babies with MS/MS up until 1 July 2019, has identified 311 infants with one of the diseases (incidence 1:3200), which is essentially the same as in other countries. The PPV for the whole period has been 47%, the sensitivity 98.73%, and the specificity 99.96%. The false positive rate has been 0.035% and the false negative rate 1.27%. At the beginning of screening, the PPV was relatively high because we already used the R4S tool and has improved further with the implementation of the CLIR analytic tools and second-tier analyses for the evaluation of results; now the PPV is 58%. This combined approach has resulted in a high performing Swedish newborn screening program. The R4S and CLIR databases and the continuous support from the R4S/CLIR team have been instrumental for these results.

## Figures and Tables

**Table 1:** All recalls, true positive cases, incidences during and before screening, false positive cases, missed cases, and PPV values during the entire reporting period. Within parenthesis are listed the corresponding numbers if counting babies with confirmed vitamin B12 deficiency as false positive. N/A stands for not applicable.

Group	Disease	All Recalls	True Positive Cases	Incidence	Incidence before Screening	False Positive Cases	Missed Cases	PPV
Amino acidemias	HCY	15	3	1:330,000	1:265,000	12	-	20%
	MSUD	20	9	1:110,000	1:265,000	11	-	45%
	PKU	77	74 ^2^	1:14,000	N/A	3	-	96%
Carnitine disorders	CACT/CPT2	14	3 ^3^	1:330,000	N/A	11	2 CPT2 ^6^	21%
	CUD	107	13	1:80,000	N/A	94 ^5^	-	12%
	CPT1	6	5	1:200,000	N/A	1	-	83%
Fatty acid oxidation defects	LCHAD	14	13	1:80,000	1:92,000	1	-	93%
	MAD	39	5	1:170,000	N/A	34	-	15%
	MCAD	67	59	1:17,000	1:235,000	8	-	88%
	VLCAD	46	24	1:42,000	1:1,060,000	22	-	52%
Organic acidurias	BKT	2	2	1:500,000	N/A	0	-	100%
	GA1	31	9	1:110,000	1:235,000	22	-	29%
	IVA	18	6	1:170,000	1:530,000	12	-	33%
	MMA/PA	165	63 (15) ^4^	1:16,000 (1:67,000)	N/A (1:118,000)	102 (150)	1 PA ^7^	38% (9%)
	TYR	10	9	1:110,000	N/A	1	-	90%
Urea cycle disorders	ARG	4	2	1:500,000	1:265,000	2	-	50%
	ASA ^1^	5	3	1:330,000	N/A	2	1 ASA ^8^	60%
	CIT ^1^	25	9	1:110,000	1:2,120,000	16	-	36%
Total		665	311 (263)	1:3200 (1:3800)	N/A	354 (402)	4	47% (40%)

^1^ We could not separate ASA and CIT at point of recall before 2015. ^2^ Three had 6-pyruvoyl-tetrahydropterin synthase deficiency. ^3^ One CACT and two CPT2, one of which also had MAD. ^4^ Four methylmalonyl-coenzyme A mutase deficiency, one cobalamin A deficiency, three cobalamin C deficiency, seven PA and 48 B12 deficiency. ^5^ Six were due to confirmed maternal CUD and six due to confirmed maternal carnitine-lowering medication. ^6^ One was from 2011, the other was from 2012. ^7^ From 2014. ^8^ From 2016.

**Table 2:** Impact of second-tier analysis on false positive cases and PPV values. Babies with confirmed B12 deficiency are included. Within parenthesis are the numbers when babies with vitamin B12 deficiency are counted as false positives.

	Before or after Second-Tier	Time Period	All Recalls	True Positive Cases	False Positive Cases	PPV	Number of Screened Newborns in Period
**IVA**	Before	15 Nov. 2010–08 Dec. 2010	11	0	11	0%	7334
	After	09 Dec. 2010–1 Jul. 2019	7	6	1	86%	993,000
**HCY**	Before	15 Nov. 2010–31 Dec. 2016	13	1	12	8%	706,543
	After	1 Jan. 2017–1 Jul. 2019	2	2	0	100%	293,791
**MMA/PA**	Before	15 Nov. 2010–31 Dec. 2016	139	47 (8)	92 (131)	34% (6%)	706,543
	After	1 Jan. 2017–1 Jul. 2019	26	16 (7)	10 (19)	62% (27%)	293,791

## Abbreviations

ARG	Arginase deficiency
ASA	Argininosuccinic aciduria
BKT	Betaketothiolase deficiency
CACT	Carnitine acylcarnitine translocase deficiency
CIT	Citrullinemia type 1
CLIR	Collaborative laboratory integrated reports
CPT1	Carnitine palmitoyl transferase 1 deficiency
CPT2	Carnitine palmitoyl transferase 2 deficiency
CUD	Carnitine uptake deficiency
DBS	Dried blood spots
GA1	Glutaric aciduria type 1
HCY	Homocystinuria
IVA	Isovaleric aciduria
LCHAD	Long-chain 3-hydroxyacyl-coenzyme A dehydrogenase deficiency
LIS	Laboratory information system
MAD	Multiple acyl-coenzyme A dehydrogenase deficiency
MCAD	Medium-chain acyl-coenzyme A dehydrogenase deficiency
MMA	Methylmalonic aciduria
MS/MS	Tandem mass spectrometry
MSUD	Maple syrup disease
N/A	Not applicable
PA	Propionic aciduria
PKU	Phenylketonuria
PPV	Positive predictive value
R4S	Region 4 Stork
TYR	Tyrosinemia type 1
VLCAD	Very long-chain acyl-coenzyme A dehydrogenase deficiency
